# Crystal structure of (*E*)-4-{2-[4-(all­yloxy)phen­yl]diazen­yl}benzoic acid

**DOI:** 10.1107/S1600536814023745

**Published:** 2014-11-15

**Authors:** Md. Lutfor Rahman, Mashitah Mohd. Yusoff, Jamil Ismail, Huey Chong Kwong, Ching Kheng Quah

**Affiliations:** a University Malaysia Pahang, Faculty of Industrial Sciences and Technology, 26300 Gambang, Kuantan, Pahang, Malaysia; b School of Chemical Sciences, Universiti Sains Malaysia, 11800 USM, Penang, Malaysia; c X-ray Crystallography Unit, School of Physics, Universiti Sains Malaysia, 11800 USM, Penang, Malaysia

**Keywords:** crystal structure, azo­benzene, benzoic acid, liquid crystal, nematic phase

## Abstract

The title compound, C_16_H_14_N_2_O_3_, has an *E* conformation about the azo­benzene [—N=N– = 1.2481 (16) Å] linkage. The benzene rings are almost coplanar [dihedral angle = 1.36 (7)°]. The O atoms of the carb­oxy­lic acid group are disordered over two sets of sites and were refined with an occupancy ratio of 0.5:0.5. The two disordered components of the carb­oxy­lic acid group make dihedral angles of 1.5 (14) and 3.8 (12)° with the benzene ring to which they are attached. In the crystal, mol­ecules are linked *via* pairs of O—H⋯O hydrogen bonds, forming inversion dimers. The dimers are connected *via* C—H⋯O hydrogen bonds, forming ribbons lying parallel to [120]. These ribbons are linked *via* C—H⋯π inter­actions, forming slabs parallel to (001).

## Abstract 2

The title compound has an *E* conformation about the azo­benzene linkage and the benzene rings are almost coplanar to one another [dihedral angle = 1.36 (7)°]. In the crystal, a combination of O—H⋯O and C—H⋯O hydrogen bonds and C—H⋯π inter­actions leads to the formation of slabs parallel to (001).

## Chemical context   

It is inter­esting to note that the title compound shows a nematic phase (Cr 190 N 218 I) . Hence, liquid crystallinity may be induced by the formation of hydrogen-bonded dimers. A number of liquid crystal (LC) systems containing hydrogen bonds that function between identical mol­ecules have been reported (Kang & Samulski, 2000 ▶; Rahman *et al.*, 2012 ▶). Much attention has been paid to hydrogen-bonded supra­molecular LCs, including LC dimers based on hydrogen-bonding inter­actions and several supra­molecular LC trimers based on hydrogen-bonding inter­actions (Lee *et al.*, 2001 ▶; Paleos & Tsiourvas, 2001 ▶; Takahashi *et al.*, 2003 ▶; Bai *et al.*, 2007 ▶). A particular aspect of photonics, in which the mol­ecular geometry can be controlled by light, is being proposed as a future technology for optical storage devices (Ikeda & Tsutsumi, 1995 ▶; Jayalaxmi *et al.*, 2009 ▶). The heart of the phenomenon in such systems is the reversible photo-induced shape transformation of the mol­ecules containing the photochromic azo­benzene groups. The title compound contains an azo (—N=N—) linkage, it was easy to synthesize and hence cost-effective for the possibility of photochromism and photoisomerization usage (Lutfor *et al.*, 2013**a* ▶,b* ▶). We report herein on its synthesis and crystal structure.
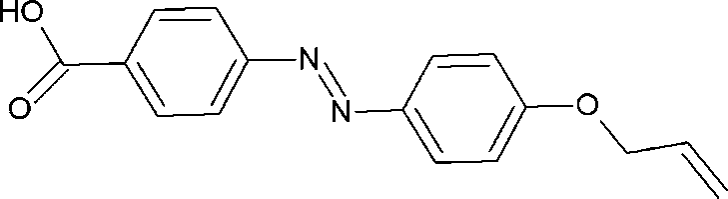


## Structural commentary   

**Figure 1:**
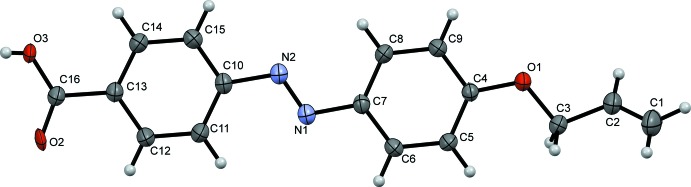
The mol­ecular structure of the title compound, showing the atom labelling. Displacement ellipsoids are drawn at the 30% probability level. Only one component of the disordered carb­oxy­lic acid group is shown.

The mol­ecular structure of the title mol­ecule is illustrated in Fig. 1 ▶. The oxygen atoms forming the carb­oxy­lic acid group are each disordered over two positions and were refined with half occupancy. The carb­oxy­lic acid group (C16/O2/O3) is almost coplanar with the attached benzene ring (C10–C15), making dihedral angles of 3.44 (9) and 3.65 (8)° for the two disorder components. The title compound has an *E* conformation about the azo­benzene (—N=N—) linkage, the length of the N1—N2 bond is 1.2481 (16) Å and the torsion angle for the azo unit (C7—N1=N2—C10) is 179.99 (10)°, which is comparable with the values of *ca* ±180° observed in 4,4-azinodi­benzoic acid (Yu & Liu, 2009 ▶) and (*E*)-ethyl-4-{[4-(deca­noxl­oxy)phen­yl]diazenly} benzoate (Lai *et al.*, 2002 ▶). The benzene rings (C4–C9) and (C10–C15) are almost coplanar, making a dihedral angle of 1.38 (7)°, compared with 6.79 (9)° in the previously reported compound 4-{(*E*)-2-[4-(but-3-en-1-yl­oxy)phen­yl]-diazen-1-yl}benzoic acid, (Rahman *et al.*, 2012 ▶).

## Supra­molecular features   

**Figure 2:**
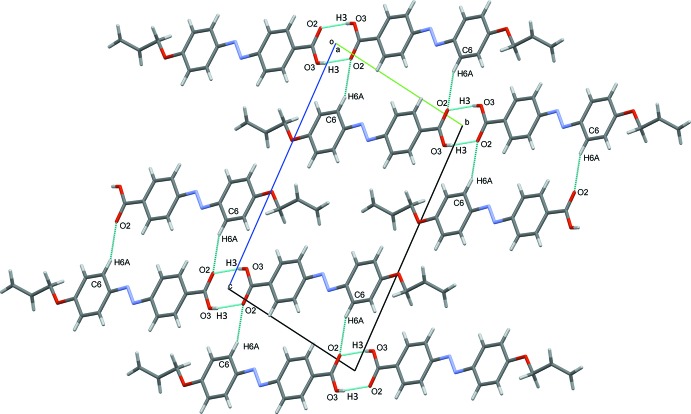
A partial view along the *a*-axis of the crystal packing of the title compound, with hydrogen bonds shown as dashed lines (see Table 1 ▶ for details).

**Figure 3:**
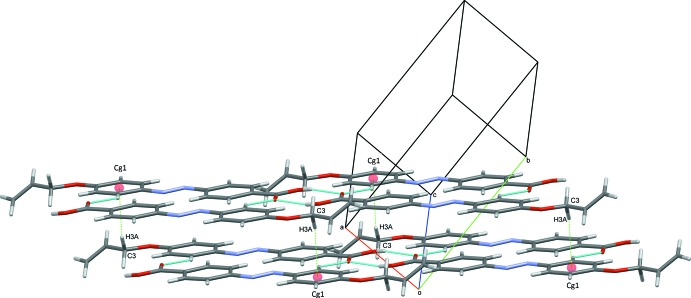
A partial view of the crystal packing of the title compound. Blue dashed lines represent the inter­molecular hydrogen bonds within two-mol­ecule-thick chains and the green dashed lines represent the weak inter­molecular C—H⋯π inter­actions (see Table 1 ▶ for details).

In the crystal, mol­ecules are linked *via* pairs of O—H⋯O hydrogen bonds, forming inversion dimers (Table 1 ▶ and Fig. 2 ▶). The dimers are connected *via* C—H⋯O hydrogen bonds, forming two-mol­ecule-thick ribbons lying parallel to [120]; see Table 1 ▶ and Fig. 3 ▶. Adjacent ribbons are linked *via* C—H⋯π inter­actions, forming slabs parallel to (001), as shown in Fig. 3 ▶ (Table 1 ▶).

## Synthesis and crystallization   

The title compound was synthesized by a literature procedure (Rahman *et al.*, 2012 ▶). The diazo­nuim salt was prepared with sodium nitrite and subsequent coupling with phenol to afforded the ethyl 4-[(4-hy­droxy­phen­yl)diazen­yl]benzoate, which was purified by crystallization and recrystallization from methanol. The azo­benzene compound was alkyl­ated with allyl bromide to give ethyl 4-{[4-(all­yloxy)phen­yl]diazen­yl}benzoate, which was purified by crystallization from methanol/chloro­form**.** The terminal double bonds-containing azo­benzene compound was hydrolysed under basic conditions to yield the title compound. Red plate-like crystals were obtained by crystallization from an ethanol–ethyl acetate mixture (1:1); m.p. 494 K. ^1^H NMR (CDCl_3_): δ 8.18 (*d*, 2H, *J* = 8.2 Hz), 7.94 (*d*, 2H, *J* = 7.1 Hz), 7.93 (*d*, 2H, *J* = 6.7 Hz), 7.05 (*d*, 2H, *J* = 8.9 Hz), 6.04 (*m*, 1H, CH=), 5.45 (*d*, 1H, *J* = 16.6 Hz, =CH_2_), 5.31 (*d*, 1H, *J* = 10.2 Hz, =CH_2_), 4.60 (*d*, 2H, *J* = 4.1 Hz, OCH_2_).

## Refinement   

Crystal data, data collection and structure refinement details are summarized in Table 2 ▶. Atoms O2 and O3 of the carb­oxy­lic acid group are each disordered over two positions and were refined with half occupancy each. The position of the O-bound H atom was located in a difference Fourier map and refined as a riding atom: O—H = 0.82 Å with *U*_iso_(H) = 1.5 *U*_eq_(O). The C-bound H atoms were positioned geometrically and refined using a riding model: C—H = 0.93–0.97 Å with *U*_iso_(H) = 1.2*U*_eq_(C). Two outlier reflections, 341 and 309, were omitted from the refinement.

## Supplementary Material

Crystal structure: contains datablock(s) I. DOI: 10.1107/S1600536814023745/su2790sup1.cif

Structure factors: contains datablock(s) I. DOI: 10.1107/S1600536814023745/su2790Isup2.hkl

Supporting information file. DOI: 10.1107/S1600536814023745/su2790Isup3.cml

CCDC reference: 1031374

Additional supporting information:  crystallographic information; 3D view; checkCIF report

## Figures and Tables

**Table 1:** Hydrogen-bond geometry (, ) *Cg*1 is the centroid of the C4C9 ring.

*D*H*A*	*D*H	H*A*	*D* *A*	*D*H*A*
O3H3O2^i^	0.82	1.90	2.71(3)	166
C6H6*A*O2^ii^	0.93	2.59	3.367(15)	145
C3H3*A* *Cg*1^iii^	0.97	2.66	3.504(2)	145

Symmetry codes: (i) 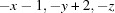; (ii) 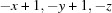; (iii) 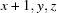.

**Table 2:** Experimental details

Crystal data	
Chemical formula	C_16_H_14_N_2_O_3_
*M*_r_	282.29
Crystal system, space group	Triclinic, *P* 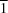
Temperature (K)	294
*a*, *b*, *c* ()	5.0279(4), 8.9678(7), 15.9913(13)
, , ()	80.571(2), 83.874(2), 88.371(2)
*V* (^3^)	707.19(10)
*Z*	2
Radiation type	Mo *K*
(mm^1^)	0.09
Crystal size (mm)	0.78 0.22 0.09

Data collection	
Diffractometer	Bruker *APEX* DUO CCD area detector
Absorption correction	Multi-scan (*SADABS*; Bruker, 2009 ▶)
*T*_min_, *T*_max_	0.931, 0.992
No. of measured, independent and observed [*I* > 2(*I*)] reflections	12171, 3276, 2344
*R*_int_	0.023
(sin /)_max_ (^1^)	0.651

Refinement	
*R*[*F* ^2^ > 2(*F* ^2^)], *wR*(*F* ^2^), *S*	0.043, 0.138, 1.04
No. of reflections	3276
No. of parameters	211
H-atom treatment	H-atom parameters constrained
_max_, _min_ (e ^3^)	0.24, 0.16

Computer programs: *APEX2* and *SAINT* (Bruker, 2009 ▶), *SHELXS97*, *SHELXL97* and *SHELXTL* (Sheldrick, 2008 ▶), *Mercury* (Macrae *et al.*, 2008 ▶), and *PLATON* (Spek, 2009 ▶).

## supplementary crystallographic information

### Crystal data

**Table:**

C_16_H_14_N_2_O_3_	*Z* = 2
*M**_r_* = 282.29	*F*(000) = 296
Triclinic, *P*1	*D*_x_ = 1.326 Mg m^−^^3^
Hall symbol: -P 1	Melting point: 494 K
*a* = 5.0279 (4) Å	Mo *K*α radiation, λ = 0.71073 Å
*b* = 8.9678 (7) Å	Cell parameters from 3848 reflections
*c* = 15.9913 (13) Å	θ = 2.5–27.4°
α = 80.571 (2)°	µ = 0.09 mm^−^^1^
β = 83.874 (2)°	*T* = 294 K
γ = 88.371 (2)°	Plate, red
*V* = 707.19 (10) Å^3^	0.78 × 0.22 × 0.09 mm

### Data collection

**Table:**

Bruker APEX DUO CCD area-detector diffractometer	3276 independent reflections
Radiation source: fine-focus sealed tube	2344 reflections with *I* > 2σ(*I*)
Graphite monochromator	*R*_int_ = 0.023
φ and ω scans	θ_max_ = 27.6°, θ_min_ = 1.3°
Absorption correction: multi-scan (*SADABS*; Bruker, 2009)	*h* = −6→6
*T*_min_ = 0.931, *T*_max_ = 0.992	*k* = −11→11
12171 measured reflections	*l* = −20→20

### Refinement

**Table:**

Refinement on *F*^2^	Primary atom site location: structure-invariant direct methods
Least-squares matrix: full	Secondary atom site location: difference Fourier map
*R*[*F*^2^ > 2σ(*F*^2^)] = 0.043	Hydrogen site location: inferred from neighbouring sites
*wR*(*F*^2^) = 0.138	H-atom parameters constrained
*S* = 1.04	*w* = 1/[σ^2^(*F*_o_^2^) + (0.0696*P*)^2^ + 0.0848*P*] where *P* = (*F*_o_^2^ + 2*F*_c_^2^)/3
3276 reflections	(Δ/σ)_max_ < 0.001
211 parameters	Δρ_max_ = 0.24 e Å^−^^3^
0 restraints	Δρ_min_ = −0.16 e Å^−^^3^

### Special details

**Table:**

Geometry. All e.s.d.'s (except the e.s.d. in the dihedral angle between two l.s. planes) are estimated using the full covariance matrix. The cell e.s.d.'s are taken into account individually in the estimation of e.s.d.'s in distances, angles and torsion angles; correlations between e.s.d.'s in cell parameters are only used when they are defined by crystal symmetry. An approximate (isotropic) treatment of cell e.s.d.'s is used for estimating e.s.d.'s involving l.s. planes.
Refinement. Refinement of *F*^2^ against ALL reflections. The weighted *R*-factor *wR* and goodness of fit *S* are based on *F*^2^, conventional *R*-factors *R* are based on *F*, with *F* set to zero for negative *F*^2^. The threshold expression of *F*^2^ > σ(*F*^2^) is used only for calculating *R*-factors(gt) *etc*. and is not relevant to the choice of reflections for refinement. *R*-factors based on *F*^2^ are statistically about twice as large as those based on *F*, and *R*-factors based on ALL data will be even larger.

### Fractional atomic coordinates and isotropic or equivalent isotropic displacement parameters (Å^2^)

**Table:**

	*x*	*y*	*z*	*U*_iso_*/*U*_eq_	Occ. (<1)
O1	1.26119 (18)	−0.00858 (10)	0.39744 (5)	0.0464 (3)	
O2	−0.234 (5)	0.8701 (17)	−0.0212 (8)	0.068 (3)	0.50
O3	−0.424 (5)	0.940 (2)	0.1072 (12)	0.0525 (17)	0.50
H3	−0.5052	1.0045	0.0769	0.079*	0.50
O2X	−0.392 (5)	0.943 (2)	0.0945 (13)	0.062 (3)	0.50
O3X	−0.267 (5)	0.8749 (16)	−0.0231 (8)	0.065 (3)	0.50
H3X	−0.3393	0.9554	−0.0404	0.098*	0.50
N1	0.6027 (2)	0.37818 (12)	0.20184 (7)	0.0442 (3)	
N2	0.4446 (2)	0.45802 (12)	0.24161 (7)	0.0444 (3)	
C1	1.6508 (4)	−0.33243 (19)	0.43299 (12)	0.0807 (6)	
H1A	1.5953	−0.3863	0.3931	0.097*	
H1B	1.7412	−0.3818	0.4775	0.097*	
C2	1.6019 (3)	−0.18848 (16)	0.42651 (9)	0.0538 (4)	
H2A	1.6599	−0.1378	0.4674	0.065*	
C3	1.4597 (3)	−0.09989 (14)	0.35815 (8)	0.0437 (3)	
H3A	1.5844	−0.0364	0.3182	0.052*	
H3B	1.3758	−0.1671	0.3273	0.052*	
C4	1.1092 (2)	0.08615 (13)	0.34590 (7)	0.0373 (3)	
C5	1.1259 (3)	0.09639 (14)	0.25801 (7)	0.0421 (3)	
H5A	1.2504	0.0384	0.2297	0.051*	
C6	0.9539 (3)	0.19454 (14)	0.21297 (8)	0.0438 (3)	
H6A	0.9626	0.2013	0.1541	0.053*	
C7	0.7698 (3)	0.28242 (13)	0.25406 (8)	0.0395 (3)	
C8	0.7558 (3)	0.27159 (14)	0.34268 (8)	0.0434 (3)	
H8A	0.6323	0.3302	0.3709	0.052*	
C9	0.9240 (3)	0.17465 (14)	0.38793 (8)	0.0436 (3)	
H9A	0.9147	0.1678	0.4468	0.052*	
C10	0.2754 (3)	0.55478 (13)	0.18998 (8)	0.0412 (3)	
C11	0.2795 (3)	0.56604 (17)	0.10240 (9)	0.0572 (4)	
H11A	0.3976	0.5065	0.0729	0.069*	
C12	0.1078 (3)	0.66589 (17)	0.05898 (9)	0.0579 (4)	
H12A	0.1103	0.6733	0.0002	0.069*	
C13	−0.0685 (3)	0.75530 (14)	0.10282 (8)	0.0427 (3)	
C14	−0.0730 (3)	0.74195 (15)	0.19032 (8)	0.0463 (3)	
H14A	−0.1914	0.8010	0.2201	0.056*	
C15	0.0973 (3)	0.64144 (15)	0.23369 (8)	0.0455 (3)	
H15A	0.0919	0.6322	0.2926	0.055*	
C16	−0.2538 (3)	0.86292 (15)	0.05719 (8)	0.0461 (3)	

### Atomic displacement parameters (Å^2^)

**Table:**

	*U*^11^	*U*^22^	*U*^33^	*U*^12^	*U*^13^	*U*^23^
O1	0.0458 (5)	0.0547 (5)	0.0370 (4)	0.0192 (4)	−0.0066 (4)	−0.0037 (4)
O2	0.062 (4)	0.097 (5)	0.039 (3)	0.042 (3)	−0.008 (2)	0.009 (3)
O3	0.052 (3)	0.062 (3)	0.041 (5)	0.029 (2)	0.000 (3)	−0.009 (3)
O2X	0.067 (7)	0.076 (3)	0.041 (4)	0.039 (4)	−0.006 (4)	−0.009 (3)
O3X	0.071 (6)	0.079 (4)	0.045 (3)	0.045 (3)	−0.012 (2)	−0.012 (3)
N1	0.0475 (6)	0.0424 (6)	0.0412 (6)	0.0075 (5)	−0.0088 (5)	−0.0013 (4)
N2	0.0459 (6)	0.0442 (6)	0.0423 (6)	0.0092 (5)	−0.0089 (5)	−0.0032 (4)
C1	0.1015 (15)	0.0604 (10)	0.0731 (11)	0.0305 (10)	−0.0072 (10)	0.0038 (8)
C2	0.0522 (8)	0.0555 (8)	0.0526 (8)	0.0157 (7)	−0.0097 (6)	−0.0056 (6)
C3	0.0425 (7)	0.0438 (7)	0.0446 (6)	0.0098 (5)	−0.0055 (5)	−0.0081 (5)
C4	0.0359 (6)	0.0389 (6)	0.0359 (6)	0.0044 (5)	−0.0062 (5)	−0.0020 (5)
C5	0.0418 (7)	0.0462 (7)	0.0372 (6)	0.0100 (5)	−0.0021 (5)	−0.0067 (5)
C6	0.0482 (8)	0.0497 (7)	0.0324 (6)	0.0071 (6)	−0.0062 (5)	−0.0031 (5)
C7	0.0411 (7)	0.0372 (6)	0.0391 (6)	0.0038 (5)	−0.0081 (5)	−0.0011 (5)
C8	0.0464 (7)	0.0426 (6)	0.0410 (6)	0.0123 (5)	−0.0040 (5)	−0.0088 (5)
C9	0.0472 (7)	0.0497 (7)	0.0339 (6)	0.0105 (6)	−0.0060 (5)	−0.0072 (5)
C10	0.0414 (7)	0.0389 (6)	0.0425 (6)	0.0045 (5)	−0.0097 (5)	−0.0014 (5)
C11	0.0620 (9)	0.0640 (9)	0.0432 (7)	0.0305 (7)	−0.0043 (6)	−0.0079 (6)
C12	0.0668 (10)	0.0678 (9)	0.0360 (6)	0.0298 (8)	−0.0064 (6)	−0.0039 (6)
C13	0.0418 (7)	0.0426 (7)	0.0421 (6)	0.0094 (5)	−0.0067 (5)	−0.0028 (5)
C14	0.0465 (8)	0.0483 (7)	0.0448 (7)	0.0132 (6)	−0.0061 (6)	−0.0110 (5)
C15	0.0492 (8)	0.0498 (7)	0.0383 (6)	0.0073 (6)	−0.0093 (5)	−0.0076 (5)
C16	0.0440 (8)	0.0490 (7)	0.0436 (7)	0.0149 (6)	−0.0046 (6)	−0.0048 (6)

### Geometric parameters (Å, º)

**Table:**

O1—C4	1.3613 (14)	C5—C6	1.3868 (17)
O1—C3	1.4317 (15)	C5—H5A	0.9300
O2—C16	1.238 (13)	C6—C7	1.3812 (18)
O3—C16	1.36 (2)	C6—H6A	0.9300
O3—H3	0.8200	C7—C8	1.3989 (17)
O2X—C16	1.18 (2)	C8—C9	1.3700 (18)
O3X—C16	1.280 (14)	C8—H8A	0.9300
O3X—H3X	0.8200	C9—H9A	0.9300
N1—N2	1.2481 (16)	C10—C15	1.3781 (18)
N1—C7	1.4195 (16)	C10—C11	1.3855 (19)
N2—C10	1.4254 (16)	C11—C12	1.3817 (19)
C1—C2	1.297 (2)	C11—H11A	0.9300
C1—H1A	0.9300	C12—C13	1.3899 (18)
C1—H1B	0.9300	C12—H12A	0.9300
C2—C3	1.4790 (19)	C13—C14	1.3825 (18)
C2—H2A	0.9300	C13—C16	1.4813 (18)
C3—H3A	0.9700	C14—C15	1.3801 (18)
C3—H3B	0.9700	C14—H14A	0.9300
C4—C5	1.3870 (16)	C15—H15A	0.9300
C4—C9	1.3956 (17)		
			
C4—O1—C3	117.90 (9)	C8—C9—C4	120.20 (11)
C16—O3—H3	109.5	C8—C9—H9A	119.9
C16—O3X—H3X	109.5	C4—C9—H9A	119.9
N2—N1—C7	114.00 (10)	C15—C10—C11	119.92 (12)
N1—N2—C10	114.63 (10)	C15—C10—N2	114.92 (11)
C2—C1—H1A	120.0	C11—C10—N2	125.15 (12)
C2—C1—H1B	120.0	C12—C11—C10	119.87 (12)
H1A—C1—H1B	120.0	C12—C11—H11A	120.1
C1—C2—C3	124.08 (15)	C10—C11—H11A	120.1
C1—C2—H2A	118.0	C11—C12—C13	120.24 (12)
C3—C2—H2A	118.0	C11—C12—H12A	119.9
O1—C3—C2	107.64 (10)	C13—C12—H12A	119.9
O1—C3—H3A	110.2	C14—C13—C12	119.40 (12)
C2—C3—H3A	110.2	C14—C13—C16	119.70 (11)
O1—C3—H3B	110.2	C12—C13—C16	120.89 (11)
C2—C3—H3B	110.2	C15—C14—C13	120.30 (12)
H3A—C3—H3B	108.5	C15—C14—H14A	119.9
O1—C4—C5	124.57 (11)	C13—C14—H14A	119.9
O1—C4—C9	115.09 (10)	C10—C15—C14	120.24 (12)
C5—C4—C9	120.33 (11)	C10—C15—H15A	119.9
C6—C5—C4	118.92 (11)	C14—C15—H15A	119.9
C6—C5—H5A	120.5	O2X—C16—O2	123.9 (14)
C4—C5—H5A	120.5	O2X—C16—O3X	117.6 (15)
C7—C6—C5	121.13 (11)	O2—C16—O3X	8 (2)
C7—C6—H6A	119.4	O2X—C16—O3	7.0 (19)
C5—C6—H6A	119.4	O2—C16—O3	128.6 (14)
C6—C7—C8	119.40 (11)	O3X—C16—O3	121.9 (13)
C6—C7—N1	116.43 (11)	O2X—C16—C13	120.1 (11)
C8—C7—N1	124.16 (11)	O2—C16—C13	115.8 (10)
C9—C8—C7	120.02 (11)	O3X—C16—C13	122.3 (9)
C9—C8—H8A	120.0	O3—C16—C13	115.6 (9)
C7—C8—H8A	120.0		
			
C7—N1—N2—C10	−179.99 (10)	C15—C10—C11—C12	1.0 (2)
C4—O1—C3—C2	178.33 (11)	N2—C10—C11—C12	−179.04 (13)
C1—C2—C3—O1	132.68 (17)	C10—C11—C12—C13	0.1 (3)
C3—O1—C4—C5	2.60 (19)	C11—C12—C13—C14	−0.9 (2)
C3—O1—C4—C9	−178.51 (11)	C11—C12—C13—C16	179.98 (14)
O1—C4—C5—C6	178.13 (11)	C12—C13—C14—C15	0.4 (2)
C9—C4—C5—C6	−0.70 (19)	C16—C13—C14—C15	179.59 (12)
C4—C5—C6—C7	0.6 (2)	C11—C10—C15—C14	−1.5 (2)
C5—C6—C7—C8	−0.3 (2)	N2—C10—C15—C14	178.60 (11)
C5—C6—C7—N1	−179.63 (11)	C13—C14—C15—C10	0.7 (2)
N2—N1—C7—C6	−178.28 (11)	C14—C13—C16—O2X	4.5 (14)
N2—N1—C7—C8	2.47 (19)	C12—C13—C16—O2X	−176.3 (14)
C6—C7—C8—C9	0.1 (2)	C14—C13—C16—O2	178.8 (10)
N1—C7—C8—C9	179.33 (12)	C12—C13—C16—O2	−2.0 (11)
C7—C8—C9—C4	−0.2 (2)	C14—C13—C16—O3X	−176.7 (11)
O1—C4—C9—C8	−178.46 (11)	C12—C13—C16—O3X	2.5 (11)
C5—C4—C9—C8	0.5 (2)	C14—C13—C16—O3	−1.5 (10)
N1—N2—C10—C15	178.98 (11)	C12—C13—C16—O3	177.7 (10)
N1—N2—C10—C11	−1.0 (2)		

### Hydrogen-bond geometry (Å, º)

Cg1 is the centroid of the C4–C9 ring.

**Table:**

*D*—H···*A*	*D*—H	H···*A*	*D*···*A*	*D*—H···*A*
O3—H3···O2^i^	0.82	1.90	2.71 (3)	166
C6—H6*A*···O2^ii^	0.93	2.59	3.367 (15)	145
C3—H3*A*···*Cg*1^iii^	0.97	2.66	3.504 (2)	145

Symmetry codes: (i) −*x*−1, −*y*+2, −*z*; (ii) −*x*+1, −*y*+1, −*z*; (iii) *x*+1, *y*, *z*.
